# Exploratory analysis of livestock waste treatment impacts on microbial diversity and antimicrobial resistance gene abundance

**DOI:** 10.1128/spectrum.01476-26

**Published:** 2026-07-15

**Authors:** Jade Davies, Jessica Ireland-Hughes, Silvia Stronati, Richard P. Smith, Claire Oastler, Javier Nunez-Garcia, Muna F. Anjum, Manal AbuOun

**Affiliations:** 1 Department of Bacteriology, Animal and Plant Health Agency 16232, Weybridge, United Kingdom; 2 Department of Epidemiology, Animal and Plant Health Agency 16232, Weybridge, United Kingdom; Connecticut Agricultural Experiment Station, New Haven, Connecticut, USA

**Keywords:** anaerobic digestion, microbiology, slurry lagoon, slurry, waste management, shotgun metagenomics, AMR

## Abstract

The potential spread of antimicrobial resistance (AMR) through agricultural waste is underexplored and may contribute to the dissemination of AMR genes into the environment. This pilot study used metagenomic sequencing to investigate how anaerobic digestion (AD) and on‐farm slurry lagoon treatment affect microbial community composition and relative AMR gene abundance in livestock waste. Samples were collected before and after treatment from three AD sites and two on-farm slurry lagoon sites. Taxonomic profiles and diversity metrics were generated from short-read Illumina sequencing, and AMR gene presence and relative abundance were assessed using APHA SeqFinder, an in-house analysis pipeline. AD treatment led to decreased microbial richness and evenness, and reduced the relative abundance of several high-prevalence taxa, including members of the Enterobacteriaceae. On-farm slurry lagoon treatment had a comparatively minor effect on microbial composition. AD was also associated with significant reductions in the relative abundance of genes conferring resistance to macrolides, aminoglycosides, fusidic acid, and beta-lactams. These findings suggest that AD and on-farm slurry lagoon treatment exert distinct effects on microbial communities and AMR gene profiles. The results provide preliminary evidence that AD may contribute to reducing AMR gene burden in agricultural waste, although further investigation across broader temporal scales and treatment methods is needed.

**IMPORTANCE:**

Antimicrobial resistance is a major global health challenge, and agricultural waste is a key environmental reservoir of resistance genes. This study examined how two livestock waste treatments (anaerobic digestion and on-farm slurry lagoon storage) affect microbial communities and relative antimicrobial resistance gene (ARG) abundance. The findings show that anaerobic digestion reduces both microbial diversity and the relative abundance of several resistance genes, while on-farm slurry lagoon treatment has a limited impact. These results highlight the potential for treatment strategies to reduce the environmental spread of resistance.

## INTRODUCTION

Livestock manure represents a major component of agricultural waste streams in the UK, with over 72 million tonnes collected annually for land application and a further 73 million tonnes deposited directly onto land in England and Wales (1). While manure is widely used as an organic fertilizer for arable land due to its high nutrient content (2), it is also recognized as a reservoir of antimicrobial-resistant (AMR) bacteria, antimicrobial resistance genes (ARGs), and mobile genetic elements (MGEs) (3, 4). The land application of untreated or partially treated manure, therefore, represents a potential pathway for the dissemination of AMR into soil ecosystems, surface waters, and the food chain (5).

A range of manure management strategies is employed to mitigate these risks, including long-term storage in slurry lagoons, composting, and anaerobic digestion (AD) (6). These processes can substantially alter microbial community structure and function through physicochemical changes, such as temperature shifts, redox conditions, nutrient availability, and microbial competition (7, 8). In particular, AD is widely implemented as a waste-to-energy technology and has been shown to restructure microbial communities toward anaerobic consortia dominated by fermentative and methanogenic taxa (9, 10). However, its impact on ARG dynamics is less clear (11). Longitudinal studies of UK dairy slurry systems have demonstrated that manure stores can act as dynamic reservoirs of ARGs and antimicrobial-resistant bacteria, with population stability maintained through continuous input of fresh waste (3).

Previous studies have produced mixed findings, with some demonstrating reductions in ARG abundance following treatment, particularly with extended storage or thermophilic conditions, while others report persistence or enrichment of specific ARG classes despite reductions in microbial load. For instance, a UK study on dairy slurry storage demonstrated that storing slurry without additional input for at least 60 days reduced the abundance of ARGs before land application (3). In contrast, a study in China reported that anaerobic digestion of pig manure did not significantly reduce the ARG burden (12). Furthermore, UK-based studies investigating the impact of AD on ARG abundance have focused on human sewage rather than agricultural waste (13, 14). These inconsistencies are likely driven by differences in feedstock composition, operational parameters (e.g., temperature, retention time, and substrate composition), and the distinction between absolute and relative abundance measures (9–11). Furthermore, ARGs associated with MGEs may persist independently of shifts in taxonomic composition, reflecting the role of horizontal gene transfer and MGE-mediated dissemination in maintaining ARGs across changing microbial communities (15). Changes in ARG abundance have also been linked to shifts in microbial community composition, with evidence that bacterial succession during treatment processes can influence ARG profiles through changes in host taxa (11).

Despite a growing body of literature on manure treatment and AMR, several knowledge gaps remain. First, much of the existing work has been conducted outside the UK, limiting its applicability to UK farming systems, which differ in livestock management practices and regulatory frameworks. Furthermore, studies often utilize controlled laboratory systems, such as bioreactors, which offer enhanced experimental control but may not reflect real-world variability and conditions; therefore, this study utilizes on-farm sampling.

Many previous studies have focused on targeted approaches, such as 16S rRNA gene sequencing, or earlier “next-generation” sequencing technologies, such as 454 pyrosequencing, which limit taxonomic resolution and do not capture functional potential (9, 15). Furthermore, studies often focus on either microbial community composition or ARG profiles in isolation, rather than integrating both to understand how ecological shifts relate to resistome dynamics (3). Additionally, there is limited comparative data across commonly used on-farm treatment strategies under real-world conditions, particularly for slurry lagoon storage versus AD, where reported ARG reduction efficiencies vary widely depending on system design and operating conditions (16). Finally, relatively few studies utilize shotgun metagenomics despite its ability to resolve both taxonomic composition and functional gene content, including ARGs and MGEs (3, 16).

To address these gaps, this pilot study aimed to provide an initial, on-farm assessment of microbial community and resistome changes following slurry storage and AD, recognizing that sample sizes are limited but sufficient to identify trends and inform larger-scale investigations. Understanding these dynamics is crucial not only for environmental protection but also for informing best practices for farmers, supporting industry standards, and guiding evidence-based policy development (17). Metagenomic sequencing was used to profile microbial communities and resistance genes, providing a powerful approach to assess how different waste treatment strategies influence the resistome (18, 19). We hypothesized that waste treatment processes would alter the metagenomic composition of farm waste, leading to a decrease in the prevalence of ARGs and a reduction in the relative abundance of *Escherichia* and *Enterococcus. Escherichia* and *Enterococcus* spp. were specifically considered given their relevance as indicator microorganisms for AMR (3, 20, 21), as well as being fecal indicator bacteria (22) and including clinically relevant species associated with human and animal infections (20, 23). To test this, samples were collected before and after treatment from three AD sites and two on-farm slurry lagoon sites, four of which processed cattle waste and one of which processed pig waste.

## MATERIALS AND METHODS

### Site selection and sampling

Sites were selected to enable a structured comparison of waste treatment approaches while controlling, as far as feasible, for waste source. An initial list of candidate sites was identified through engagement with the Environment Agency, industry bodies (REAL Schemes, ADBA), and academic collaborators, with selection criteria including (i) treatment of predominantly single-animal waste streams to reduce confounding from mixed inputs, and (ii) representation of treatment types of interest, specifically AD and storage.

The study was designed to include paired comparisons within dairy systems, which represented most of the available sites. To facilitate this, two dairy AD sites and two dairy slurry storage (lagoon) sites were selected. AD sites were further chosen to be broadly comparable in operational parameters (e.g., predominantly dairy slurry input, on-farm waste sourcing, mesophilic conditions ~40°C, propeller mixing, and similar treatment durations of 21–28 days), enabling treatment effects to be assessed with reduced process heterogeneity. Two dairy lagoon sites were selected to provide a corresponding storage-based comparison. In addition, one pig AD site was included to extend the analysis to an alternative livestock system while maintaining a predominantly single-species waste stream.

Sites were recruited on a voluntary basis, and the final selection reflects a pragmatic subset of those willing and able to participate within the project timeframe. The broader study initially targeted 10 sites (balanced across pig and cattle systems) to assess antimicrobial-resistant *Escherichia coli* using culture-based approaches; however, resource constraints limited metagenomic analysis to five sites. As such, the selected sites represent a targeted, comparative design rather than a statistically representative sample of all UK waste treatment systems.

Sampling was conducted according to a standardized protocol provided to site operators, where waste samples were collected from each site before and after treatment, with two replicate samples collected at each time point. Each replicate comprised a composite (pooled) sample intended to capture within-site heterogeneity.

For AD sites, pre-treatment samples were taken from mixed waste immediately prior to entry into the AD tank. Post-treatment samples were collected from digestate at the point of removal from the tank, prior to storage or land application. Where present, both liquid and solid fractions were sampled. For slurry lagoons, a composite sampling approach was used. Pre-storage samples were obtained from fresh waste entering the lagoon by combining subsamples collected from different animal groups or housing areas contributing to the waste stream. These subsamples were pooled to generate two composite replicate samples per site.

Post-storage samples were collected during lagoon emptying. Subsamples were taken at multiple stages of the emptying process (i.e., at the start, in the middle, and at the end) to account for spatial and temporal variability within the lagoon and were pooled to produce two composite replicate samples. At all sites, sampling was performed using clean equipment and gloves. Sample containers were filled approximately halfway, sealed, stored at 2°C–8°C, and couriered overnight to the laboratory.

### DNA sequencing and data quality control

DNA extraction was performed on 200 mg of each sample using the GenFind HMW DNA kit (Beckman Coulter Life Sciences, UK). Library preparation and short-read sequencing were performed using an Illumina NovaSeq 6000 instrument with 2  × 150 bp configuration using a sequencing depth of 5 Gb per sample (Novogene, UK).

Raw reads were processed to remove host contamination (cow and pig genomes) using Bowtie2 (v2.5.2) (24). Sequencing adaptors were removed using fastp (v0.23.4), and reads were trimmed from the 3′ end using an average sliding four-base minimum Phred score of Q20, and a minimum read length of 80 bp (25). Taxonomic classification was performed with Kraken2 (v.2.1.3) (26) with a confidence threshold set at 0.1 (27) and using a custom 540 GB database of 241,589 genomes, including GTDB R220 representatives and selected plant and fungal genomes (accessed 13/12/2024). Taxa contributing ≤0.0005% of hits per sample were filtered out (28). Raw abundance values were imported into RStudio (2024.12.0) and normalized using DESeq2 (v1.48.1) (29) prior to analysis and visualization using R (v4.5.0) (30). Summary statistics for metagenomic read quality and yield are provided in Table S10.

### Data analysis and visualization

To evaluate the impact of waste treatment, we applied three complementary analyses: alpha diversity (within-sample diversity), beta diversity (between-sample compositional dissimilarity), and ARG profiling. Alpha diversity metrics (species richness, Shannon index, and Simpson index) were calculated using the vegan R package (v2.7.1) (31). These metrics were derived from genus-level taxonomic relative abundance tables generated from metagenomic classification. The Simpson index of diversity is presented as 1 – D, where values closer to 1 indicate higher diversity and values closer to 0 indicate lower diversity.

For beta diversity analysis, taxonomic relative abundance data were aggregated at the genus level and used to construct Bray–Curtis dissimilarity matrices using the vegdist function in vegan (method = “bray”) (31). Genus-level aggregation was used to reduce sparsity in the data and improve the interpretability of compositional differences across samples. Prior to analysis, genera with total normalized abundance <20 across all samples were removed, and the top 30 most abundant genera were visualized using heatmaps generated with ggplot2 (v3.5.2) (32), pheatmap (v1.0.13) (33), and tidyverse (v2.0.0) (32, 34). Principal coordinate analysis (PCoA) plots were created using the cmdscale function in base R (30). In this study, “time point” refers to the sampling stage relative to treatment, i.e., pre-treatment (incoming waste) versus post-treatment (treated material). Relationships between metadata categories (time point, site type, and site) and principal component (PC) scores were explored using non-parametric statistical tests. Additionally, permutational multivariate analysis of variance (PERMANOVA) was performed on Bray–Curtis dissimilarities using the adonis2 function in the vegan package (31). Wilcoxon rank-sum tests were employed for comparisons between two groups (site type, time point), and Kruskal-Wallis tests for comparisons among three or more groups (site). *P* values were adjusted for multiple hypothesis testing using Benjamini–Hochberg correction, and a false discovery rate threshold of <0.05 was used to determine statistically significant differences (35, 36).

### Detection of AMR genes

Host-depleted, unfiltered reads were taken as input to APHA SeqFinder (37). SeqFinder is routinely used with reads and assemblies from isolates (38), but was modified for use with metagenomic reads. APHA SeqFinder first performs quality checking via fastp and then uses both SMALT (39) to detect acquired AMR genes using an in-house reference database comprising 2,890 genes across 21 antimicrobial classes (40, 41) using a 70% minimum identity threshold to allow detection of gene variants. The criteria for determining gene presence using APHA SeqFinder was 70% of the query gene mapping to the reference with no gaps or non-calls, allowing up to 100 SNP variants (synonymous and/or non-synonymous) (42).

Relative gene abundance was quantified by calculating the absolute number of hits for each gene using the formula: mapped length multiplied by sequencing depth, divided by the read length. To normalize for sequencing depth and enable comparisons across samples and conditions, the absolute hits were then converted to a proportional measure by dividing the absolute hits by the total number of reads in the sample and multiplying by one million, yielding the number of gene hits per million reads. Genes with a lower 95% confidence interval (L95CI) at or below zero proportional hits per million reads were excluded, as their presence could not be reliably confirmed. Among these genes with a lower bound of the 95% confidence interval (L95CI) equal to zero, the maximum upper 95% confidence interval (U95CI) was identified and used as a threshold. Genes with L95CI values below this threshold were subsequently excluded to ensure higher confidence in gene presence. Genes with >10 absolute hits were retained. The final set of genes, expressed as proportion of hits per million metagenomic reads, was used for downstream visualization and comparative analysis. Visualization was conducted using the R packages ggplot2 and pheatmap. Differentially abundant AMR genes were identified using unpaired Wilcoxon rank-sum tests for two-group comparisons and Kruskal-Wallis tests for comparisons among three or more groups. *P* values were adjusted using the Benjamini–Hochberg correction. Genes with raw *P* values ≤ 0.05 were displayed on heatmaps using the R packages ggplot2 and pheatmap with relative abundance values log-transformed for interpretability.

## RESULTS

### Microbial diversity

**Fig 1:**
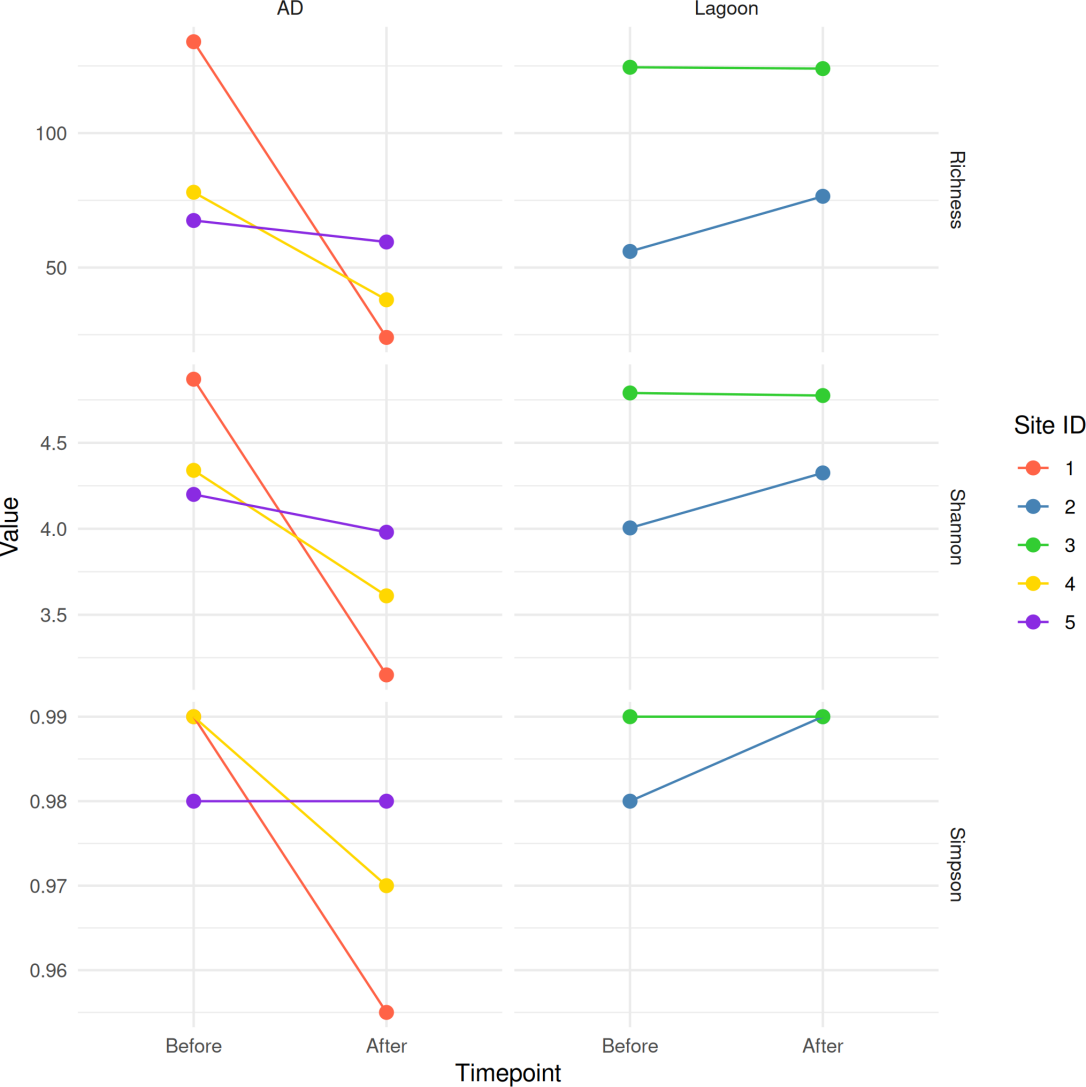
Alpha diversity of microbial communities before and after waste treatment across five agricultural sites. Observed richness, Shannon diversity, and Simpson diversity indices are shown for each site, with values derived from shotgun metagenomic sequencing at the genus level. Points represent mean values calculated from two replicates, with lines connecting paired samples collected before and after treatment at each site. Data are shown separately for AD and on-farm slurry lagoon systems (lagoon). Simpson index is presented as 1 − D, where values closer to 1 indicate higher diversity.

Alpha diversity trends varied across sites and treatment types. At AD sites 1 and 4, where the waste source was from pig and cattle, respectively, we observed lower genera richness and diversity (Shannon and Simpson indices) after treatment (Fig. 1; Table 2), indicating a shift in community composition. In contrast, AD site 5, also from the cattle waste source, maintained stable diversity scores before and after treatment (Fig. 1; Table 2). In comparison, among the on-farm slurry lagoon sites, site 2 showed an increase in diversity following treatment, while site 3 remained unchanged (Fig. 1; Table 2). Overall, the AD sites displayed a trend of reduced richness and diversity of bacterial genera post-treatment, a pattern not observed in the on-farm slurry lagoon sites (Fig. 1; Table 2). Notably, all metagenomes had consistently high Simpson indices, indicating low dominance by individual genera and a relatively even distribution of abundances across taxa.

### Microbial community composition

**Fig 2:**
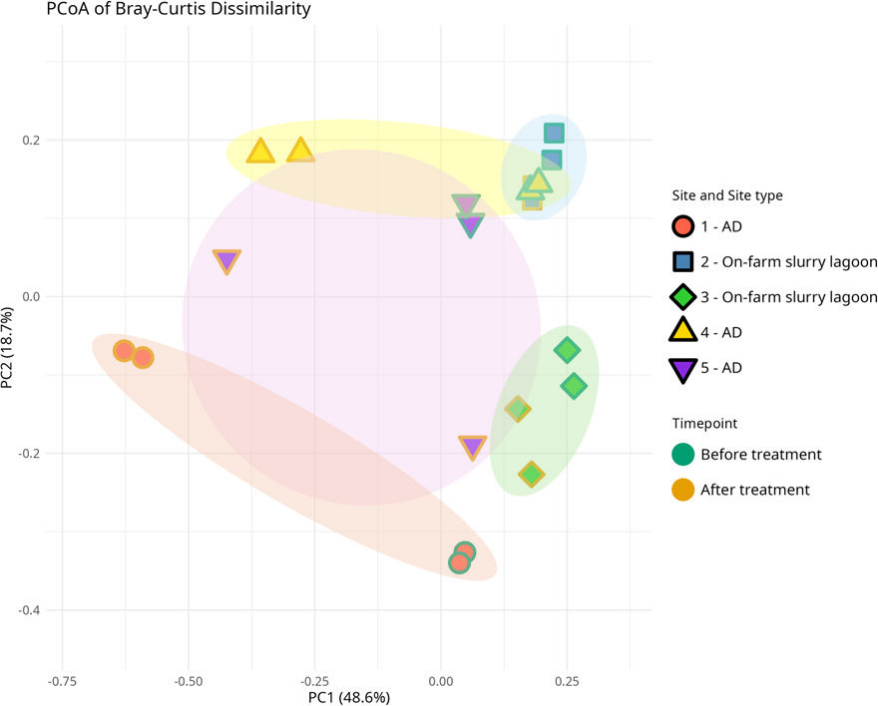
PCoA plot of samples based on Bray–Curtis dissimilarity scores. Each point represents a sample, and ellipses are colored by site. The axes represent the first two principal components (PC1 and PC2).

PCoA based on Bray–Curtis dissimilarities indicated separation in microbial community composition between sites and treatment systems, with visual shifts observed at AD sites following treatment (Fig. 2). Changes at on-farm slurry lagoon sites were less pronounced (Fig. 2). PERMANOVA confirmed that site, site type, and time point all significantly contributed to variation in microbial community composition (Table S12). Site type explained the largest proportion of variation (R² = 0.238, *P* = 0.001), followed by time point (R² = 0.167, *P* = 0.001), and site (R² = 0.127, *P* = 0.008). These results indicate that while differences between treatment systems are the primary drivers of community composition, shifts associated with treatment stage (pre- versus post-treatment) are also detectable, albeit more modest in magnitude.

**Fig 3:**
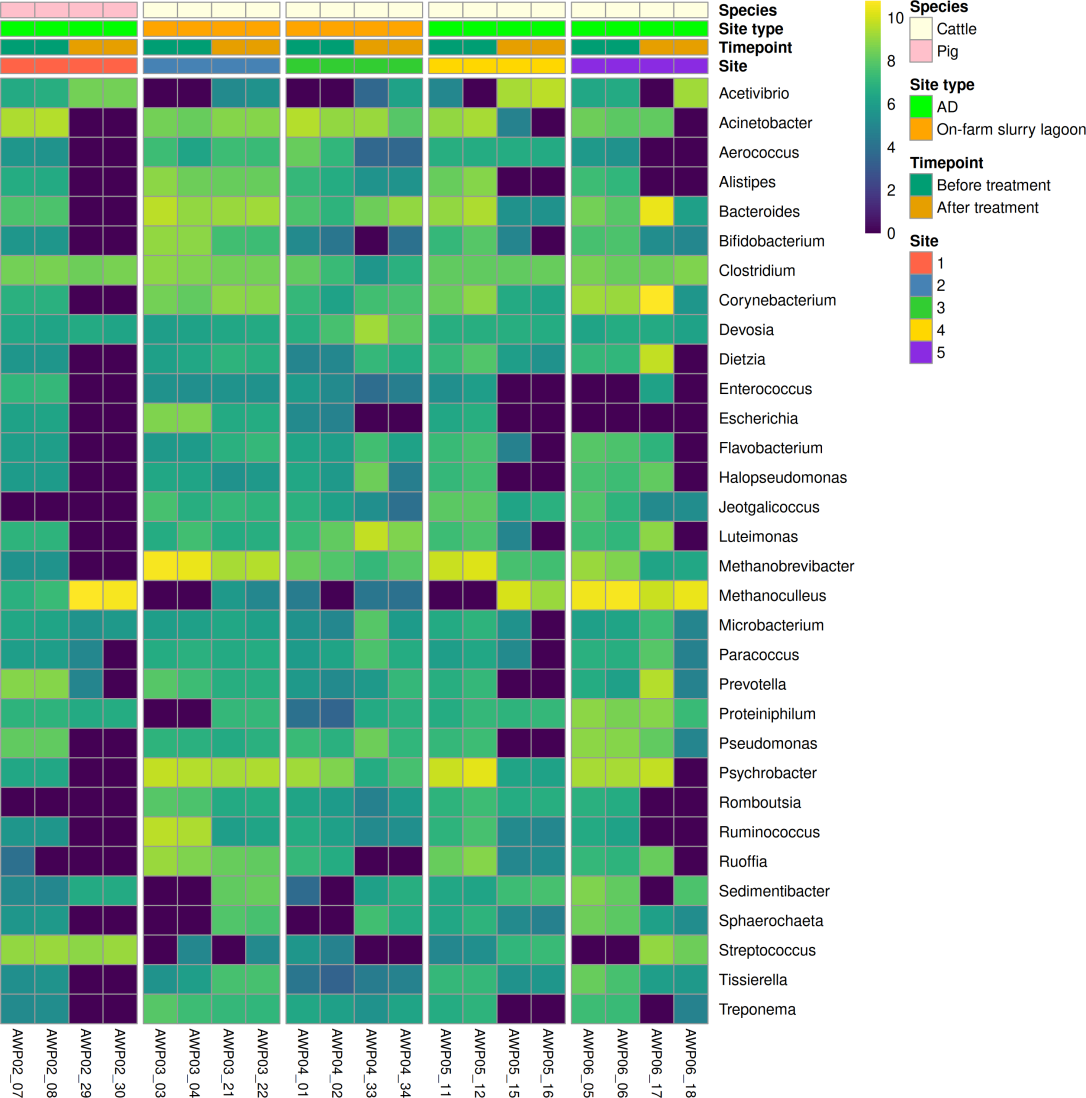
Heatmap showing relative abundance of the top 30 most abundant genera in addition to *Enterococcus* and *Escherichia*. Rows represent genera, and columns represent sample conditions.

A marked loss of several of the 30 most highly abundant bacterial genera was observed on site 1 (AD) due to the waste treatment, including *Acinetobacter*, *Aerococcus*, *Alistipes*, *Bacteroides*, *Bifidobacterium*, *Corynebacterium*, *Dietzia*, *Flavobacterium*, *Halospseudomonas*, *Luteimonas*, *Methanobrevibacter*, *Pseudomonas*, *Psychrobacter*, *Ruminococcus*, *Sphaerochaeta*, *Tissierella*, and *Treponema* (Fig. 3). This loss was accompanied by a decrease in genera richness (Table 2). Conversely, at the on-farm slurry lagoon sites (sites 2 and 3), increases in the relative abundance of specific genera were observed after waste treatment. Increased relative abundance of *Acetivibrio*, *Sedimentibacter*, and *Sphaerochaeta* was observed at both on-farm slurry lagoon sites post-treatment (Fig. 3). Additionally, on site 2, there was increased proportional abundance of *Methanoculleus* and *Proteiniphilum*, and site 3 had higher *Streptococcus* proportional abundance post-treatment (Fig. 3). Both *Escherichia* and *Enterococcus* decreased in relative abundance post-treatment at two AD sites (1 and 4) (Fig. 3). However, *Escherichia* was not detected in metagenomic samples from AD site 5, and only one post-treatment sample from this site contained *Enterococcus* (Fig. 3). Conversely, the on-farm slurry lagoon sites did not exhibit clear treatment-related effects on these genera; while *Escherichia* relative abundance decreased post-treatment at site 3, it remained unchanged at site 2, and *Enterococcus* relative abundance was unaffected at both sites (Fig. 3).

### Impact of time point (before versus after treatment) on microbial profiles

**Fig 4:**
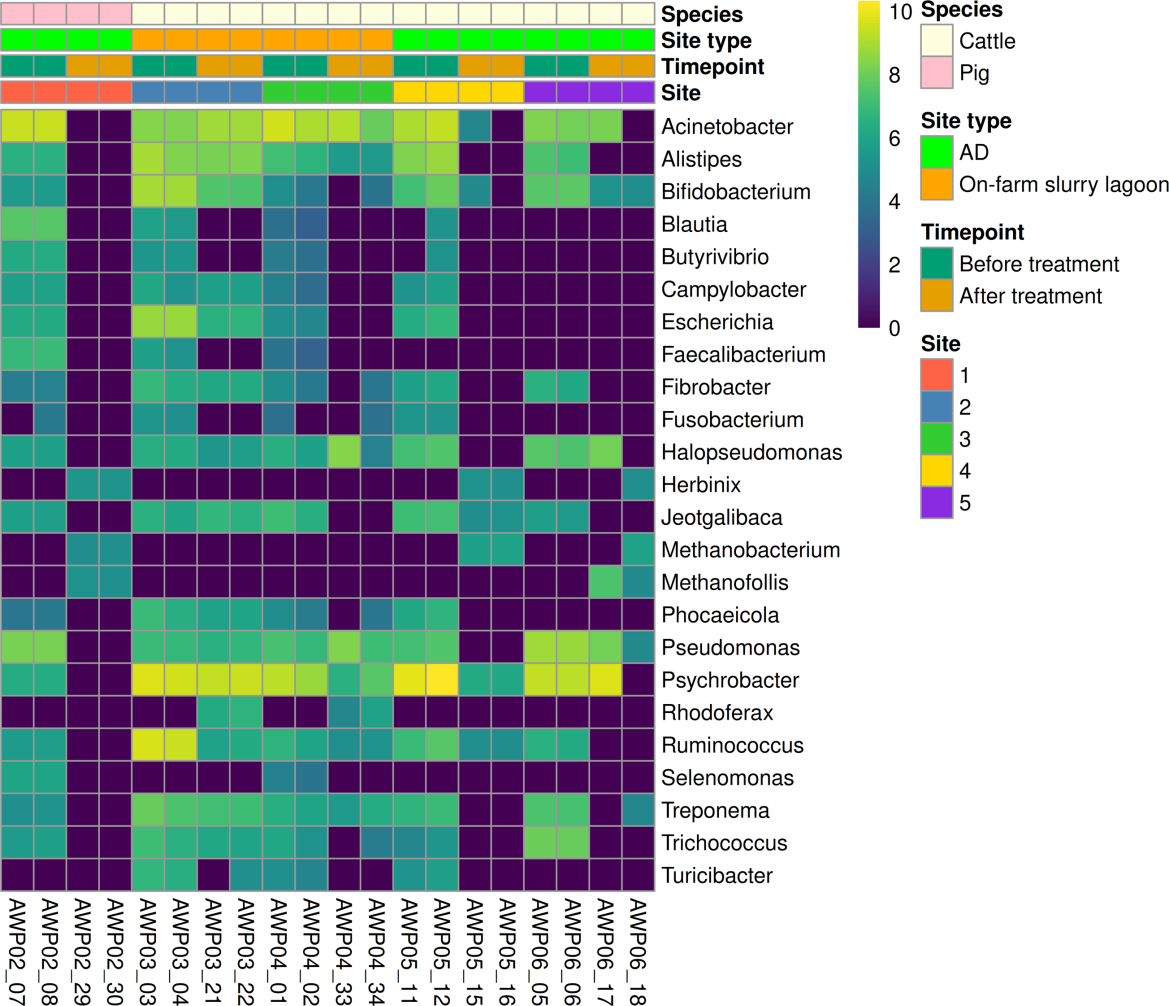
Heatmap showing relative abundance of genera with raw *P* values ≤ 0.05 from differential abundance testing by time point. Rows represent genera, and columns represent sample conditions.

Differential abundance testing, comparing microbial profiles before and after waste treatment, did not reveal any genera with statistically significant changes in relative abundance after correcting for multiple hypothesis testing (Table S2). However, 24 genera showed trends toward significance, defined here as raw *P* values ≤ 0.05 prior to multiple hypothesis adjustment (Table S2; Fig. 4). Many of these genera were among the top 30 most abundant across the data set, including *Acinetobacter*, *Alistipes*, *Bifidobacterium*, *Escherichia*, *Halopseudomonas*, and *Treponema* (Fig. 3), suggesting that their loss post-treatment was likely to have been biologically relevant. Clinically relevant genera, including *Escherichia* and *Campylobacter* (3, 20, 21, 43, 44), were lost post-treatment on AD sites 1 and 4, and lagoon site 3 (Fig. 4).

### Impact of site type (AD versus on-farm slurry lagoon) on microbial profiles

**Fig 5:**
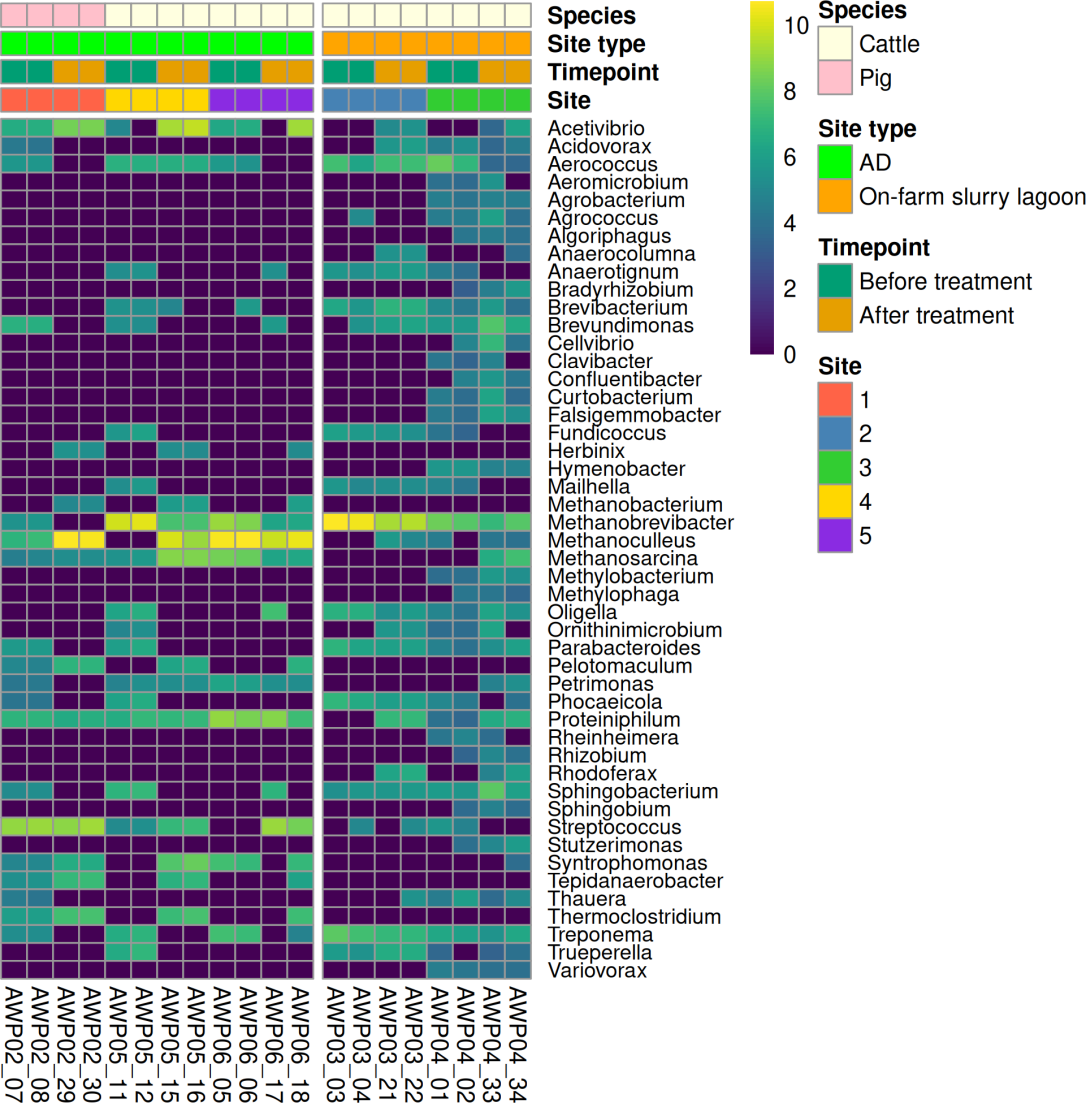
Heatmap showing relative abundance of genera with raw *P* values ≤ 0.05 from differential abundance testing by site type. Rows represent genera, and columns represent sample conditions, grouped by site type.

Differential abundance testing based on site type (AD versus on-farm slurry lagoon) did not reveal any genera that were significantly differentially abundant after multiple hypothesis testing correction (Table S3). Nonetheless, 48 genera showed trends toward statistically significant differences (raw *P* values ≤ 0.05) in relative abundance due to site type (Fig. 5). AD sites displayed increased relative abundances of genera, including *Herbinix* and *Methanobacterium,* following treatment, whereas these were not detected at on-farm slurry lagoon sites (Fig. 5). In contrast, on-farm slurry lagoon sites exhibited a post-treatment loss of *Rhodoferax*, a genus not detected in AD sites (Fig. 5). Differences between the two on-farm slurry lagoon sites were also apparent; several genera, including *Aeromicrobium*, *Agrobacterium*, *Agrococcus*, *Bradyrhizobium*, *Curtobacterium*, *Falsigemmobacter*, *Hymenobacter*, *Methylobacterium*, and *Variovorax*, were exclusively detected at farm site 3 and were absent from the other on-farm slurry lagoon site and all AD sites (Fig. 5). When testing differential abundance by site, nine genera were identified as significantly differentially abundant after correction (Table S4; Fig. S1). These differences were solely driven by the genera uniquely detected at specific sites. *Acetomicrobium*, *Caldicoprobacter*, and *Petrotoga* were exclusively found at AD site 1, while *Agrobacterium*, *Curtobacterium*, *Falsigemmobacter*, *Hymenobacter*, *Methylobacterium*, and *Variovorax* were only present at on-farm slurry lagoon site 3 (Fig. S1).

### AMR gene diversity and relative abundance

**Fig 6:**
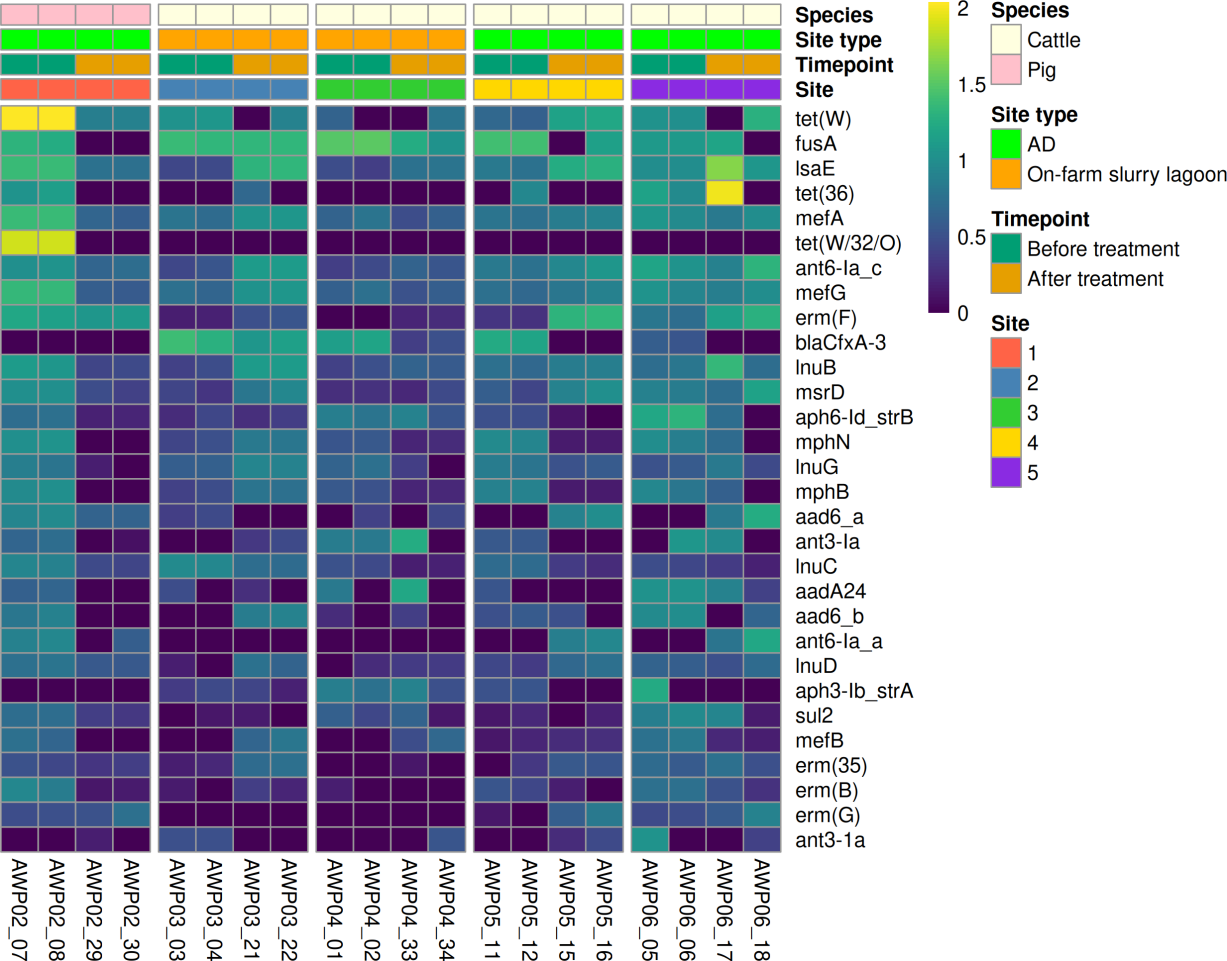
Heatmap showing log-transformed relative abundance of the 30 most abundant AMR genes. Rows represent genes, and columns represent samples.

Macrolide resistance genes were the most frequently detected class observed across the sites and time points, followed by aminoglycoside and beta-lactam resistance (Table 3). The highest number of AMR genes detected was 60 in sample AWP02_07, and the lowest was 27 in AWP02_30; the largest decrease in AMR gene counts between time points was observed on site 1, an AD site (Table 3). Table 3 summarizes AMR gene richness (number of unique genes detected per class), whereas Fig. 6 presents normalized relative abundance based on read mapping (hits per million metagenomic reads).

Among the genes analyzed, the tetracycline resistance genes *tet(W*), *tet(W/32/O*), and *tet(36*) were the most abundant (Fig. 6). Notably, *tet(W/32/O*) was detected exclusively in samples from AD site 1 before treatment and was absent from both post-treatment samples and from other sites (Fig. 6).

Differential abundance testing based on time point (before versus after waste treatment) did not reveal any AMR genes that were significantly differentially abundant after multiple hypothesis testing correction (Table S5). However, nine genes showed trends toward statistically significant differences (raw *P* values ≤ 0.05) in relative abundance due to time point (Table S5; Fig. S3). These included four genes conferring macrolide resistance [*mphB*, *mphN*, *lnuC*, *erm(Y*)], three aminoglycoside resistance genes (*aac6-Ie, aph2_If, strB*), *fusA* conferring fusidic acid resistance, and *bla*_cfxA-3_, a beta-lactam resistance gene (Fig. S3). Among these, *strB*, *fusA*, *mphB*, and *mphN* tended to decrease in relative abundance post-treatment at AD sites 1, 4, and 5, while on-farm slurry lagoon sites (2 and 3) showed little change (Fig. S3). Similarly, *bla*_cfxA-3_ decreased post-treatment at AD sites where it was initially detected (sites 4 and 5) and was absent on site 1; in contrast, *bla*_cfxA-3_ maintained relatively high abundance at on-farm slurry lagoon sites both before and after treatment (Fig. S3).

Differential abundance testing by site type revealed significant differences in the relative abundance of macrolide resistance genes [*erm(G*), *erm(F*), *erm(C*)] and the streptothricin resistance gene (*sat4A*) between AD sites and on-farm slurry lagoon sites (Fig. S4; Table S6). Three genes [*sat4A*, *erm(F*), and *erm(C*)] were detected exclusively at AD sites, whereas *erm(G*) had significantly higher relative abundance at AD sites compared to on-farm slurry lagoon sites (Fig. S4). An additional 18 genes showed trends toward statistically significant differences (raw *P* values ≤ 0.05) in relative abundance between AD sites and on-farm slurry lagoons; seven of these are macrolide resistance genes, which showed higher relative abundance in AD sites compared to on-farm slurry lagoon sites (Fig. S4; Table S6). Conversely, five beta-lactam resistance genes (*bla_CARB-14_*, *bla_CARB-49_*, *bla_CARB-B_*, *bla_CARB-8_*, *and bla_cfxA3_*) had higher relative abundance in on-farm slurry lagoon sites compared to AD sites (Fig. S4; Table S6). Differential abundance comparisons between sites did not identify any AMR genes that were significantly different after correction for multiple hypothesis testing (Table S7). However, 16 genes showed trends toward differential abundance between sites (raw *P* values ≤ 0.05). Among these were *sat4A*, *erm(F*), *erm(G*), *erm(C*), and *bla_cfxA3_* (Fig. S2). Notably, *tet(X3*), *tet(X5*), and *tet(X6*) genes conferring resistance to tigecycline, a glycylcycline antibiotic within the tetracycline class, were detected at low levels (3–4 proportional gene hits per million reads) in samples collected before treatment at two AD sites (Table S11).

## DISCUSSION

This pilot study provides preliminary insights into the effects of two livestock waste treatment methods (AD and on-farm slurry lagoon storage) on microbial community composition and AMR gene relative abundance. Shotgun metagenomic sequencing was used to assess shifts in microbial diversity and resistance profiles before and after treatment across five agricultural sites. AD exerted a more pronounced impact on microbial community composition and relative representation of resistance genes compared to on-farm slurry lagoon storage, as indicated by lower species richness and diversity indices post-treatment.

Taxonomic classification of metagenomic reads revealed distinct differences in microbial composition between AD and on-farm slurry lagoon sites. Notably, AD sites exhibited either increased or sustained relative abundance of the methanogenic genus *Methanoculleus* before and after treatment, a genus absent from on-farm slurry lagoon samples (Fig. 3). This genus, associated with marine environments and methanogenesis, has been linked to methane production during waste treatment (45) and anaerobic digestion (46, 47). Additionally, *Alistipes*, a gut-associated genus (48–50), was lost across all AD sites (Fig. 3). While *Alistipes* has previously been linked to butyric acid production in anaerobic digesters (51), its disappearance in this study could reflect species-level variation within the genus in terms of adaptability to anaerobic digester conditions.

Given the clinical relevance of Enterobacteriaceae, the relative abundance of *Escherichia* and *Enterococcus* was also examined. AD treatment resulted in a marked reduction in the proportional representation of these genera, whereas on-farm slurry lagoon storage had no detectable effect (Fig. 3). These findings provide tentative evidence that AD treatment may be more effective than on-farm slurry lagoon storage in reducing the relative abundance of potentially pathogenic and AMR-harboring bacteria. Indeed, this has been previously evidenced where psychrophilic AD maintained between 19°C and 22°C was demonstrated to reduce the abundance of *Escherichia* and *Salmonella* (52). Additionally, the microbial load and diversity within AD reactors are affected by the addition of new organic matter referred to as organic load shock, where microbial abundance decreases with increasing organic load within 72 h (53). Given that two of the AD sites had a high frequency of waste addition, i.e., either every 4 h (site 1) or every 0.5–1 h (site 5) (Table 1), this may also have contributed to the community composition shifts observed in this study.

**TABLE 1:** Descriptive characteristics of the five sites selected for metagenomic analysis^*a*^

Site type	AD	On-farm slurry lagoon	On-farm slurry lagoon	AD	AD
Site	1	2	3	4	5
Waste source	Pig	Dairy	Dairy	Dairy	Dairy
Pre-sample date	27/02/2024	14/02/2024	13/02/2024	05/03/2024	27/02/2024
Post-sample date	04/06/2024	09/04/2024	30/07/2024	26/03/2024	26/03/2024
Time between sampling (days)	98	55	168	21	28
Temperature (°C)	42	NA	NA	40	40
Type of site	AD with continuous flow	Uncovered slurry/liquid waste lagoons	Uncovered slurry/liquid waste lagoons	AD with continuous flow	AD with continuous flow
Waste source	Local farms (pig)	Own farm (dairy)	Own farm (dairy/beef)	Own farm (dairy/feed waste)	Own farm (dairy/crops)
Storage of waste material before treatment	Closed stores	Open stores	NA	Closed stores	NA
Waste storage length before treatment	Up to 1 week	4–8 weeks (weather dependent)	1 week	1 week	Continuous flow
Frequency of waste addition	Every 4 h	Several times a day	Added every 6–7 days	0.5–1 h	2 h
Mixing method	Propeller mixing	Tractor and stirrer	Farmyard manure moved and turned by telehandler	Propeller mixing	Propeller mixing
Mixing frequency	Continuous	Once every few months	Just once	Continuous	10 min every hour
Length of storage/treatment	100 days	4–8 weeks	2–10 months	21 days	28 days
Volume of waste processed (tonnes/year)	27,000	400	1,500	7,804	1,974
Final use for digestate	Crop irrigation, fertilizer	Fertilizer		Fertilizer	Fertilizer

^*a*^ Treatment duration reflects site-specific operational conditions and differs between waste treatment systems; these were not standardized or controlled experimentally.

On-farm slurry lagoon storage did not result in clear or consistent shifts in overall microbial community structure in this study, based on the alpha and beta diversity analyses performed. This is broadly consistent with previous studies demonstrating persistence of specific microbial taxa within stored agricultural waste. Wang et al. showed Shiga toxin-producing *E. coli* viability for over 125 days in dairy slurry after storage at both 4°C and 22°C (54), and *Enterococcus* spp., *Salmonella* spp., and *Clostridia* spp. were detectable after manure storage for 50 days at 20°C (55). Furthermore, *E. coli* was detected via culturing in 80% of liquid manure samples from dairy slurry lagoons taken at a single time point (56). Persistence of *Salmonella enterica* Typhimurium, *Listeria monocytogenes,* and ESBL-*E. coli* was also demonstrated in anaerobic digestate containing pig, cattle, or poultry slurry after 24 weeks of storage under four different temperatures simulating different seasons (57). The authors reported that these three pathogens do not grow but survive and persist during storage, although log reductions were observed, with the fastest reduction at the higher temperatures simulating summer and autumn seasons (57).

Twenty-four genera exhibited trends toward statistical significance (raw *P* values ≤ 0.05) when comparing microbial relative abundance before and after treatment, with these patterns largely driven by a pronounced microbial richness reduction at site 1 (Fig. 3 and 4). The divergence in microbial profiles between site 1 and the other AD sites (sites 4 and 5) may be attributable to differences in operational parameters. Specifically, site 1 operated at a slightly higher temperature (42°C versus 40°C) and had a longer interval between sampling (98 days compared to 21 and 28 days, respectively; Table 1). Furthermore, site 1 received pig waste, whereas sites 4 and 5 processed cattle waste (Table 1). These factors likely contributed to the more substantial community composition changes observed at site 1. These findings are consistent with earlier microbiome studies of anaerobic digesters, which have shown substantial heterogeneity between sites, largely driven by differences in operating conditions, such as temperature, salinity, input source, and pre-treatment methods (58). Clinically relevant genera, including *Escherichia* and *Campylobacter*, which are used as indicator organisms for fecal contamination (3, 20, 21) and AMR surveillance (43, 44) in livestock systems, were lost post-treatment at AD sites 1 and 4, as well as at lagoon site 3 (Fig. 4). *E. coli* in particular has been extensively used as an indicator organism for treatment efficiency in both wastewater and livestock waste treatment systems (59–64). This observation provides preliminary evidence that waste treatment, particularly AD, may reduce the presence of clinically significant bacteria in agricultural waste and lower the risk of environmental dissemination of AMR bacteria.

Site-specific variation in the microbiome was apparent, particularly between the two on-farm slurry lagoon sites (Table 1; Fig. S1); given that only two on-farm slurry lagoon sites were included in this analysis, and the farms varied in their operational practices, including frequency of feedstock addition, storage time prior to treatment, and the treatment length (55 and 168 days, respectively; Table S9), it is difficult to ascertain whether the observed differences can be attributed to the slurry lagoon treatment itself. This may simply reflect the higher diversity observed on site 3, which exhibited the highest Shannon diversity of the sites and consistently high species richness even post-treatment (Table 2). Further sampling of on-farm slurry lagoon sites is required to robustly assess whether these taxa are genuinely more associated with on-farm slurry lagoon sites compared to AD sites and to explore their ecological roles in waste treatment.

**TABLE 2:** Alpha diversity metrics (observed richness, Shannon, and Simpson indices) calculated from normalized microbial community data^*a*^

Site	Site type	Waste source	Time point	Richness (mean)	Shannon (mean)	Simpson (mean)
1	AD	Pig	Before	134	4.87	0.99
1	AD	Pig	After	24	3.15	0.96
2	Lagoon	Dairy	Before	56	4.01	0.98
2	Lagoon	Dairy	After	76.5	4.33	0.99
3	Lagoon	Dairy	Before	124.5	4.79	0.99
3	Lagoon	Dairy	After	124	4.78	0.99
4	AD	Dairy	Before	78	4.34	0.99
4	AD	Dairy	After	38	3.61	0.97
5	AD	Dairy	Before	67.5	4.2	0.98
5	AD	Dairy	After	59.5	3.98	0.98

^*a*^ Mean values for Shannon and Simpson diversity were calculated from two replicates.

An overall reduction in the number of AMR genes detected (ARG richness) was observed only at AD site 1 (Table 3), with additional, although less pronounced, decreases in detected gene counts noted at the other AD sites. Collectively, these patterns suggest that AD waste treatment may be more effective in reducing the ARG richness compared to on-farm slurry lagoons, potentially reflecting broader shifts in microbial community composition under AD conditions.

**TABLE 3:** Number of unique AMR genes detected (gene richness) per antimicrobial class in each sample^*a*^

Site ID	Site type	Animal species	Time point	Sample	Aminoglycoside (189)	Beta-lactam (1934)	Chloramphenicol (42)	Fusidic acid (3)	Glycoside (65)	Macrolide (105)	Streptothricin (3)	Sulfonamide (10)	Tetracycline (83)	Trimethoprim (52)	Total	Change in total AMR genes from before to after treatment
1	AD	Pig	Before	AWP02_07	11	4	1	1		26	1	2	11	3	60	↓
				AWP02_08	10	5	1	1		26	1	2	6	3	55	
			After	AWP02_29	7					18	1	1	1		28	
				AWP02_30	6					18	1	1	1		27	
2	On-farm slurry lagoon	Cattle	Before	AWP03_03	9	6		1		15			1		32	–
				AWP03_04	9	6		1		13		2	1		32	
			After	AWP03_21	7	2	1	1		20		2	1		34	
				AWP03_22	8	7	1	1	1	20		1	1		40	
3	On-farm slurry lagoon	Cattle	Before	AWP04_01	9	2	1	1		11		2	1		27	–
				AWP04_02	9	10	1	1		11		2	1		35	
			After	AWP04_33	6	8	3	1		13	1	2	1		35	
				AWP04_34	6	5	1	1	1	12		2	1		29	
4	AD	Cattle	Before	AWP05_11	10	5	1	1		19		2	2		40	–
				AWP05_12	9	3		1		17	1	2	2		35	
			After	AWP05_15	8					20	1		1		30	
				AWP05_16	7	1		1		20	1	1	1		32	
5	AD	Cattle	Before	AWP06_05	8	1	2	1		22	1	2	5		42	–
				AWP06_06	7	1	2	1		21	1	2	3		38	
			After	AWP06_17	11	2	3	1		22	2	2	6	2	51	
				AWP06_18	8	1				18	1	1	1		30	

^*a*^ Values represent gene counts, not read abundance. The number in brackets indicates the total number of genes in each class in the reference database (Table S8). Changes in total number of unique AMR genes from before to after treatment are summarised for each site using a symbol, where “↓” indicates overall decrease, “–” indicates no observable change.

Notably, genes conferring resistance to tigecycline, a glycylcycline antibiotic within the tetracycline class, were detected in samples collected before treatment at two AD sites, sites 1 and 5 (Table S11). Tigecycline is classified by the World Health Organization as a Highest Priority Critically Important Antimicrobial (65, 66) and is not approved for veterinary use (67). Specifically, *tet(X3*), *tet(X5*), and *tet(X6*) were identified (Table S11). The *tet(X*) gene encodes a flavin-dependent monooxygenase where certain variants confer resistance to tetracyclines, including tigecycline (68). The *tet(X3*), *tet(X5*), and *tet(X6*) variants share 85.5%, 89.6%, and 84.3% amino acid identity with the original *tet(X*) gene, respectively, and confer high-level resistance to tigecycline (8–32 mg/L) (69). The *tet(X*) gene variants have been detected in environmental samples, including soil from swine production facilities (70, 71) and sludge from waste treatment plants (72), and have also been reported in clinical isolates, including *Enterobacter cloacae*, *E. coli*, *Klebsiella pneumoniae,* and *Pseudomonadaceae*, in Sierra Leone (73). Due to limitations of short-read metagenomics, associating these ARGs with specific taxa was not possible. Notably, these resistance genes were only present in pre-treatment samples and were absent post-treatment (Table S11). Microbial diversity can provide an ecological context for the observed resistome patterns; however, the link between changes in microbial diversity and ARG richness could not be fully explored in this study.

It is important to emphasize that this study is based on a small number of heterogeneous, real-world treatment systems, and therefore treatment type is not independent of other operational parameters, such as retention time, feedstock composition, and loading frequency. As a result, observed differences between anaerobic digestion and slurry lagoon systems cannot be attributed solely to treatment modality, nor can the influence of individual operational variables be disentangled. Instead, the findings should be interpreted as system-level, real-world comparisons that reflect the combined effects of treatment technology and site-specific operating conditions. Accordingly, conclusions regarding the relative effectiveness of anaerobic digestion in reducing microbial diversity and ARG abundance are intended to be exploratory and hypothesis-generating rather than causal.

In summary, these findings indicate that AD treatment has a more substantial impact on microbial richness and ARG relative abundance than on-farm slurry lagoon storage, suggesting AD may be a more effective strategy for limiting environmental dissemination of ARGs. Further research is needed to validate these results as the study’s conclusions were constrained by limited replication, a small number of sites, and restricted sequencing resources. The variability between sites with respect to operating conditions and parameters (Table 1) is likely to remain a prohibitive challenge to hypothesis testing, even with larger sample sizes. Nonetheless, this study adds to the growing evidence supporting the role of waste treatment technologies in mitigating AMR spread from agricultural sources and highlights the need for broader investigations across diverse spatial and temporal contexts.

### Conclusions

Our findings suggest that AD can influence microbial community composition in agricultural waste, with observable shifts in AD sites compared to on-farm slurry lagoons. AD sites showed indications of reduced richness and diversity, shifts in community composition, and reduced relative abundances of fecal indicator bacteria, such as *Escherichia* and *Enterococcus*, whereas on-farm slurry lagoon sites remained relatively stable. Trends in the relative abundance of ARGs, including macrolide, aminoglycoside, fusidic acid, and beta-lactam resistance genes, were also observed. However, reductions in ARGs were not consistent across all AD sites, and statistical support was limited due to the small number of replicates. Taken together, these results suggest that AD has the potential to alter microbial and resistome profiles in farm waste. Further work with increased replication and sequencing depth will be required to robustly assess the extent to which AD contributes to mitigating environmental AMR risks.

## Supplementary Material

Reviewer comments

## Data Availability

The metagenomic sequencing data are publicly available under NCBI BioProject PRJNA1304896.

## SUPPLEMENTAL MATERIAL

Supplemental figures and tables spectrum.01476-26-s0001.xlsx Supplementary figures and tables.

OPEN PEER REVIEW reviewer-comments.pdf An accounting of the reviewer comments and feedback.

ASM does not own the copyrights to Supplemental Material that may be linked to, or accessed through, an article. The authors have granted ASM a non-exclusive, world-wide license to publish the Supplemental Material files. Please contact the corresponding author directly for reuse.
