# Supplementary material for: Exploratory analysis of livestock waste treatment impacts on microbial diversity and antimicrobial resistance gene abundance
Source: Microbiol Spectr. 2026 Jul 15;14(8):e01476-26. doi: 10.1128/spectrum.01476-26 (PMC13435644; doi:10.1128/spectrum.01476-26)
Supplement: Supplementary file 1 [file reviewer-comments.pdf]

AMERICAN SOCIETY FOR MICROBIOLOGY | Microbiology Spectrum

Exploratory analysis of Livestock Waste Treatment impacts on Microbial Diversity and Antimicrobial Resistance Gene Abundance

Jade Davies, Jessica Ireland-Hughes, Silvia Stronati, Richard Smith, Claire Oastler, Javier Nunez-Garcia, Muna Anjum, and Manal Abu Oun

Corresponding Author(s): Jade Davies, Animal and Plant Health Agency

Review Timeline:

Submission Date:	May 7, 2026
Editorial Decision:	May 18, 2026
Revision Received:	May 19, 2026
Accepted:	May 28, 2026

Editor: Blaire Steven

Reviewer(s): The reviewers have opted to remain anonymous.

Transaction Report:

(Note: With the exception of the correction of typographical or spelling errors that could be a source of ambiguity, letters and reports are not edited. The original formatting of letters and referee reports may not be reflected in this compilation.)

DOI: https://doi.org/10.1128/spectrum.01476-26

Re: Spectrum01476-26 (Exploratory analysis of Livestock Waste Treatment impacts on Microbial Diversity and Antimicrobial Resistance Gene Abundance)

Dear Dr. Jade Jennifer Davies:

Thank you for the privilege of reviewing your work. I am pleased to inform you that your manuscript has been editorially accepted for publication.

After having read the manuscript and response to previous review I am happy to report that I am provisionally accepting the manuscript for publication.

I would encourage the authors to verify in the journal style guide whether colored Tables are allowed. I believe with color they need to be figures, so either submit the Table as a figure or remove colors.

There are a few additional questions in the submission form that need to be answered before your manuscript is officially accepted. Once these are completed, please return your submission so that I can move your paper forward to acceptance.

Please return the manuscript within 60 days; if you cannot complete the submission questions within this time period, please contact me.

Thank you for submitting your paper to Spectrum.

Sincerely,
Blaire Steven
Editor
Microbiology Spectrum

Re: Spectrum01476-26R1 (Exploratory analysis of Livestock Waste Treatment impacts on Microbial Diversity and Antimicrobial Resistance Gene Abundance)

Dear Dr. Jade Jennifer Davies:

Your manuscript has been accepted, and I am forwarding it to the ASM production staff for publication. Your paper will first be checked to make sure all elements meet the technical requirements. ASM staff will contact you if anything needs to be revised before copyediting and production can begin. Otherwise, you will be notified when your proofs are ready to be viewed.

Data Availability: ASM policy requires that data be available to the public upon online posting of the article, so please verify all links to sequence records, if present, and make sure that each number retrieves the full record of the data. If a new accession number is not linked or a link is broken, provide production staff with the correct URL for the record. If the accession numbers for new data are not publicly accessible before the expected online posting of the article, publication may be delayed; please contact ASM production staff immediately with the expected release date.

Publication Fees: For information on publication fees and which article types have charges, please visit our website. We have partnered with Copyright Clearance Center (CCC) to collect author charges. If fees apply to your paper, you will receive a message from no-reply@copyright.com with further instructions. For questions related to paying charges through RightsLink, please contact CCC at ASM_Support@copyright.com or toll free at +1-877-622-5543. CCC makes every attempt to respond to all emails within 24 hours.

ASM Membership: Corresponding authors may join or renew ASM membership to obtain discounts on publication fees. Need to upgrade your membership level? Please contact Customer Service at Service@asmusa.org.

Embargo Policy: A press release may be issued as soon as the manuscript is posted on the Spectrum Latest Articles webpage. The corresponding author will receive an email with the subject line "ASM Journals Author Services Notification" when the article is available online.

The ASM Journals program strives for constant improvement in our submission and publication process. Please tell us how we can improve your experience by taking this quick Author Survey.

Thank you for submitting your paper to Spectrum.

Sincerely,
Blaire Steven
Editor
Microbiology Spectrum
